# Exploring Multidimensional Spatiotemporal Point Patterns Based on an Improved Affinity Propagation Algorithm

**DOI:** 10.3390/ijerph16111988

**Published:** 2019-06-04

**Authors:** Haifu Cui, Liang Wu, Zhanjun He, Sheng Hu, Kai Ma, Li Yin, Liufeng Tao

**Affiliations:** 1 Faculty of Information Engineering, China University of Geosciences, Wuhan 430074, China; cuihaifu@cug.edu.cn (H.C.); hezj@cug.edu.cn (Z.H.); husheng@cug.edu.cn (S.H.); makai@cug.edu.cn (K.M.); taoliufeng@cug.edu.cn (L.T.); 2 National Engineering Research Center of Geographic Information System, Wuhan 430074, China; 3 Department of Urban and Regional Planning, State University of New York, Buffalo, NY 14214, USA; liyin@buffalo.edu

**Keywords:** affinity propagation, spatial clustering, Gaussian kernel function, Davies-Bouldin index, trajectory points

## Abstract

Affinity propagation (AP) is a clustering algorithm for point data used in image recognition that can be used to solve various problems, such as initial class representative point selection, large-scale sparse matrix calculations, and large-scale data with fewer parameter settings. However, the AP clustering algorithm does not consider spatiotemporal information and multiple thematic attributes simultaneously, which leads to poor performance in discovering patterns from massive spatiotemporal points (e.g., trajectory points). To resolve this issue, a multidimensional spatiotemporal affinity propagation (MDST-AP) algorithm is proposed in this study. First, the similarity of spatial and nonspatial attributes is measured in Gaussian kernel space instead of Euclidean space, which helps address the multidimensional linear inseparability problem. Then, the Davies-Bouldin (DB) index is applied to optimize the parameter value of the MDST-AP algorithm, which is applied to analyze road congestion in Beijing via taxi trajectories. Experiments on different datasets and algorithms indicated that the MDST-AP algorithm can process multidimensional spatiotemporal data points faster and more effectively.

## 1. Introduction

Spatial clustering is an important data analysis technology that searches and identifies a finite set of species or clusters and then describes the spatial data. Through clustering, dense and sparse regions can be identified and then global distribution patterns and interesting relationships among the data attributes can be determined [1,2]. Spatial cluster analyses, as a branch of statistics, have been studied for many years, and they have been widely used in many fields, including urban planning, ecological environment, public health, transportation systems, and market analysis.

Spatial clustering methods are divided into partitioning methods [3,4,5], hierarchical methods [6,7,8], density-based methods [9,10,11], graph-based methods [12,13], model-based methods [14,15,16], grid-based methods [17,18,19], and other methods [20,21]. Using comparisons and analyses, these algorithms have presented certain issues, such as the choice of initial points, the setting of sensitive parameters, the global optimal solution, the independence of adjacent units, and the slow processing of large-scale data. Therefore, the selection of clustering algorithms should fully consider the clustering requirements for solving problems, which requires an improvement in existing clustering techniques and the continuous development of new theories and methods to adapt to new applications. 

With the time dimension attribute added to data mining, spatiotemporal clustering has developed. Spatiotemporal data clustering can obtain spatial distribution regularities within a sequence of events that can be used to identify hotspots and generate new space research units. Many scholars have applied spatiotemporal clustering to different fields. Nanni and Pedreschi [22] used a density-based clustering algorithm with trajectory data based on the simple notion of distance between trajectories. This approach is sensitive to the density of the dataset, and the density parameters in the algorithm can affect the quality of clustering. Birant and Kut [23] proposed an algorithm for clustering spatiotemporal data, which has the ability to discover clusters according to nonspatial, spatial, and temporal values of the objects. However, this algorithm cannot address multidimensional linear inequalities. Zhao et al. [24] presented an effective method (i.e., the graph-based clustering algorithm) to select parameters for clustering, determine the number of clusters, and identify cluster centers. However, this algorithm cannot process spatiotemporal data in a non-Euclidean space. These algorithms have achieved good clustering results for certain specific problems or fields but are not suitable for clustering multidimensional spatiotemporal data.

Spatiotemporal data generally have multidimensional and massive characteristics. However, based on the above analysis, most of the previously applied spatiotemporal data (e.g., trajectory points) clustering methods must set the initial clustering center, clustering radius, and other sensitive parameters before calculation. Different parameter settings will generate different results and many experiments cannot manage a very large amount of data with different parameters. Therefore, a clustering method is required to automatically obtain optimal solutions with few parameter settings. Moreover, the location characteristics and temporal and thematic attributes must be considered simultaneously when analyzing multidimensional spatiotemporal data to obtain accurate results.

Affinity propagation (AP) is a novel clustering algorithm that was proposed in the journal Science in 2007 [25]. Because the number of clusters does not need to be specified in advance, the problem of selecting the initial class representative points can be solved. The AP algorithm can also solve the problems associated with non-Euclidean space (e.g., not satisfying symmetry or triangle inequality) and large-scale sparse matrix calculations and quickly treat large-scale data with fewer parameter settings. Researchers have applied the AP algorithm in community structure analysis, pattern recognition, bioengineering, and other fields [26,27,28], which has led to increasing development of this algorithm. Considering the advantages of the AP algorithm, it is suitable for clustering spatiotemporal data; however, its clustering performance during inseparable multidimensional linear conditions is poor because obtaining the best clustering category automatically is difficult. Therefore, an improved multidimensional spatiotemporal affinity propagation (MDST-AP) algorithm is proposed. The MDST-AP method is appropriate for clustering analysis because it can better extract hidden information in multidimensional spatiotemporal data.

In this paper, multidimensional attributes under different scales are considered synthetically and the method of the Gaussian kernel transformation is proposed to solve the linear inseparable problem. In addition, an adaptive parameter setting method is proposed to solve the parameter setting problem of the clustering algorithm, which reduces the limitations of the artificial definition parameters. Finally, evaluations are performed with taxi trajectory data from Beijing which show that the MDST-AP algorithm can reflect traffic congestion more efficiently. The rest of the paper is arranged as follows. Section 2 introduces some related concepts for the AP algorithm. In Section 3, the MDST-AP algorithm is described in detail and Section 4 presents the clustering results with Beijing taxi data and discusses the MDST-AP algorithm performances. Section 5 provides a summary of this paper and directions for future research.

## 2. Basic Concepts

The basic idea of the AP algorithm is to use all data points as a potential cluster center (called the exemplar); then, the connection between data points forms a network (i.e., similarity matrix) and the cluster center of each sample is calculated through message (i.e., responsibility and availability) passing through each side of the network.

The AP clustering algorithm is different from the k-means algorithm or k-center algorithm as it does not need to specify the number of clusters before running the algorithm. The similarity among the calculated data points can be symmetric or asymmetric and comprise the similarity matrix $S_{N\times N}$. The s (k, k) on the diagonal of the S matrix can be regarded as the criterion for the clustering center, which means that the greater the value is, the greater the possibility that this point becomes the cluster center (also called the reference degree *P* (preference)). Therefore, the number of clusters is affected by the reference *P*. The relative definition is as follows.

**Definition** **1.** *Make dataset $X=\left\{x_{1},x_{2},\ldots ,x_{N}\right\}$ have some relatively close clustering $C=\left\{C_{1},C_{2},\ldots ,C_{K}\right\}$ (K ≤ N) in the data feature space. Each data point corresponds to only one cluster, where $x_{C\left(i\right)}\left(i=1,2,\ldots ,N\right)$ represents the representative cluster points of any point ($x_{i}$).*

The AP algorithm first considers all of the N sample points of the dataset as the candidate cluster center and establishes the attraction information for each sample point with the other sample points (i.e., the similarity between any two sample points $x_{i}$ and $x_{j}$). This similarity can be specified according to the research questions. In practical applications, Euclidean space constraints do not need to be satisfied. In traditional clustering problems, the similarity is usually set as a negative number from two Euclidean distance squares:

(1) $$s\left(i,j\right)=-d^{2}\left(x_{i},x_{j}\right)=-\|x_{i}-x_{j}{\|}^{2}\left(i\neq j\right)$$

where s(i,j) is stored in the similarity matrix $S_{N\times N}$ and indicates to what extent $x_{i}$ is suitable as the representative point of data point $x_{j}$. If $x_{i}$ is in the cluster center, the greater the attractiveness to the other data points is, the greater the possibility of becoming a cluster center; if $x_{i}$ is at the edge of the cluster, the attraction of the other points is smaller and the possibility of becoming a cluster center is reduced. Before clustering, the algorithm sets the bias parameter s(i,i) for each point $x_{i}$. The greater the value of s(i,i) is, the greater the probability of the selected corresponding point $x_{i}$ as a class representative point. The size of the *P* value is an important parameter in the AP algorithm, as it affects the number of final clusters. The larger *P* is the more data points that tend to become final class representative points and the greater the number of final clusters. Conversely, if *P* is smaller, the number of final output clusters is less. Therefore, the AP algorithm can find the appropriate cluster number by changing the *P* value. Generally, *P* is set as the median of the similarity matrix.

The AP algorithm continuously searches for two different types of information for selecting the appropriate cluster center: the “responsibility” information and the “availability” information, where the two types of information represent different competitive purposes. r(i,j) represents the attractiveness information, which is used to represent the degree of representation of $x_{j}$ as the class representative point of $x_{i}$. a(i,j) represents the degree of belonging information, which is used to represent the degree of suitability of data point $x_{i}$ and selected data point $x_{j}$ as a representative of its class. The greater r(i,j) and a(i,j) are, the greater the possibility of point $x_{j}$ being the final cluster center. Each sample point obtains the final clustering center through repeated iterative competition. The iterative process of the AP algorithm is the process of alternation and renewal of these two types of information. At the initial stage of the algorithm, both r(i,j) and a(i,j) are set to 0, and the following are the formulas for r and a:

(2) $$r\left(i,j\right)=s\left(i,j\right)-\underset{k\neq j}{\max}\left[a\left(i,k\right)+s\left(i,k\right)\right]$$

(3) $$a\left(i,j\right)=\{\begin{array}{c} \min\left\{0,r\left(j,j\right)+\underset{k\neq i,k\neq j}{\sum }\max\left[0,r\left(k,j\right)\right]\right\},i\neq j\\ \underset{k\neq j}{\sum }\max\left[0,r\left(k,j\right)\right],i=j \end{array}$$

From Equations (2) and (3), when s(k,k) is larger, r(k,k) is larger, and a(i,j) is larger; therefore, the class representative K is more likely to be the final cluster center. Similarly, the greater s(k,k) is the more class representatives tend to become the final cluster center. Therefore, increasing or decreasing s(k,k) can increase or decrease the number of AP output clusters.

In addition, the AP algorithm introduces an important parameter called the damping factor. In each cycle iteration, the updated results of r(i,j) and a(i,j) are weighted by the updated values in the current iterative process and the previous iteration results, which avoids numerical oscillation in the iterative process. Among them, 0≤λ≤1, with the default value being 0.5. When the number of classes generated in the AP algorithm is constantly oscillating and cannot converge, the increase in λ can eliminate damping.

Based on an in-depth analysis of the AP algorithm and combined with the practical application requirements, this study includes attribute dimension, distance measurement, similarity value and other aspects. The MDST-AP algorithm is proposed, as it can fit the data distribution structure accurately.

## 3. Methodology

**Figure 1:**
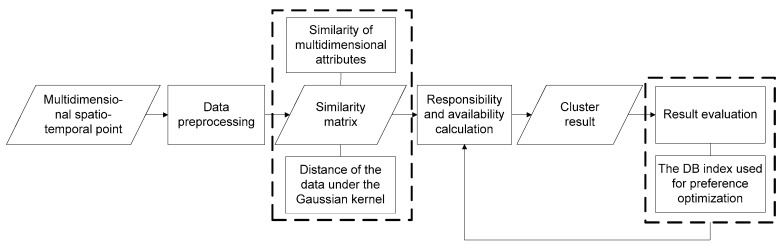
Flow chart of the multidimensional spatiotemporal affinity propagation (MDST-AP) algorithm.

The MDST-AP clustering analysis procedure is presented in Figure 1: data processing, similarity matrix establishment, cluster validity evaluation, and responsibility and availability calculation. The detailed calculation process was presented by the pseudocode of the MDST-AP algorithm in Section 3.5.

The data preprocessing refers to data selection, map matching and normalization. Based on the experimental requirements, the necessary data are kept and erroneous data are removed. The shortest distance method is used to match the trajectory points to the road network. To ensure that data with different units or orders of magnitude remain comparable, appropriate changes to the data are usually required. This paper uses the Z-score normalization method, which is applicable to situations in which the maximum and minimum values of attributes are unknown or outliers beyond the range of values. After normalization, the mean value of each variable is 0 and the standard deviation is 1. The original data are all converted to a dimensionless index evaluation value in which the values of each index present the same quantity level, and then the comprehensive evaluation and analysis can be performed. “Responsibility” information and “availability” information can be calculated based on the method introduced in Section 2. Therefore, this paper focuses on performing multidimensional similarity calculation, parameter optimization and result evaluation. 

### 3.1. Similarity of Multidimensional Attributes

Traditional clustering considers only unique properties or location attributes when clustering the spatial entity; however, the proposed MDST-AP method can simultaneously calculate similarities among spatial attributes, temporal attributes, and thematic attributes. By taking the taxi trajectory point data, including the latitude and longitude, speed, and direction attributes (or more attributes), as an example, the similarity between two points is considered by the distances among all of these attribute values. The detailed calculation process is explained in Section 3.2.

### 3.2. Distance of the Data Under the Gaussian Kernel

For the linearly inseparable problem of AP clustering, the Gaussian kernel space is used in this paper. The kernel skill can provide a connection from linear to nonlinear features and represent the dot product between two vectors. If we first map the input data to a higher- dimensional space, then the effect of the operation in this high-dimensional space is nonlinear in the original space.

In many clustering applications, datasets have complex structures (e.g., multidimensional attributes, large volume, and uneven distribution). The distinct characteristic of this complex data structure is that the types of clusters are not only limited to hyperspherical density distributions but also intertwined with clusters with arbitrary shapes and different densities and the similarity relations among the objects are no longer satisfied with the traditional Euclidean space constraints. Therefore, the similarity in the absolute distance cannot fully reflect the true structural information of the dataset. In this case, the traditional AP algorithm based on the Euclidean distance measurement is no longer applicable. Starting with the improvement in the similarity measure, this paper mainly focuses on the clustering of different densities and some linear inseparable data clustering problems and proposes a concept of the similarity measure of a kernel space which effectively improves the clustering performance.

Similarity measures involve a distance measured between datasets. The AP algorithm is still a geometric distance relation based on the Euclidean space constraints, which exhibit serious defects when the dataset is linearly inseparable. According to the theory of generalized linear discriminants, the problem of linear inseparability in the input space can be transformed into a higher-dimensional feature space by constructing a suitable kernel function, which is then solved using the linear discriminant function of the characteristic space. In fact, the nonlinear dataset in the low-dimensional Euclidean space is mapped to a high-dimensional or even infinite-dimensional vector space via kernel transformation. This process brings the same types of points closer and separates different types of points, which brings the dataset closer to the linear separable case. Then, clustering is implemented in this vector space. The following concepts related to the kernel function are introduced.

**Definition** **2.** *Assume that there is a dataset $\left\{x|x\in X,X\subset R^{d}\right\}$ in a low-dimensional input space, for which nonlinear mapping is applied:*

(4) Φ:X→F,x∈X→Φ(x)∈F

Nonlinear mapping (Φ) is referred to as kernel maps. The space F is called the kernel space or the characteristic space. The original low-dimensional space X is called the sample space or the input space. After mapping the nonlinear data from the sample space to the kernel space, it is necessary to operate the new data in the kernel space, which involves the internal product operation of the vector in the kernel space. The kernel function combines the two steps of nonlinear mapping and the inner product operation of a vector in the characteristic space. This function converts the operation of the kernel space into the sample space, which makes the nonlinear mapping implicit.

**Definition** **3.** *Set $X\subset R^{d}$, where Φ represents the kernel mapping of the sample space X to kernel space F for arbitrary x,y∈X. The inner product in the kernel space 〈Φ(x),Φ(y)〉 comprises the two-variable function in the sample space, which is called the kernel function and is recorded as k(x,y),*

(5) k(x,y)=〈Φ(x),Φ(y)〉

The use of kernel functions can relieve the linear separability of the data and enhance the clustering effect, but how to design the most appropriate kernel function is difficult. In fact, the usual method is used to directly assign a kernel function with parameters and then select suitable kernel parameters by experiments or other methods. Common kernel functions can be divided into two types: local kernels and global kernels. Local kernels are divided into large-scale kernels and small-scale kernels based on the different selected kernel parameters [29].

The similarity of the Euclidean space is transformed into a kernel space; therefore, the original linear metric is transformed into a nonlinear metric (i.e., the similarity measurement in the kernel space).

**Definition** **4.** *Make $X=\left\{x_{1},x_{2},\ldots ,x_{N}\right\}$ a finite dataset of the mode space $R^{n}$; $x_{i}\left(i=1,2,\ldots ,N\right)$ is a vector in the space, and the nonlinear transformation Φ is used to map the input data space X to a high-dimensional feature space H. The high-dimensional space vector after transformation is $\Phi \left(x_{i}\right)\left(i=1,2,\ldots ,N\right)$. The distance between data points in the feature space is calculated as follows:*

(6) $$d_{H}\left(x,y\right)=\sqrt{{\left|\left|\Phi \left(x\right)-\Phi \left(y\right)\right|\right|}^{2}}=\sqrt{\Phi \left(x\right)\Phi \left(x\right)-2\Phi \left(x\right)\Phi \left(y\right)+\Phi \left(y\right)\Phi \left(y\right)}$$

The form of the dot product in the input space can be represented by a Mercer kernel in a high-dimensional feature space, which is expressed as k(x,y)=〈Φ(x),Φ(y)〉. Then, Equation (6) becomes:

(7) $$d_{H}\left(x,y\right)=\sqrt{k\left(x,x\right)-2k\left(x,y\right)+k\left(y,y\right)}$$

In the field of clustering analyses, common kernel functions are linear kernel functions, polynomial kernel functions, and the Gaussian kernel function. The linear kernel function is mainly used for linear separable cases. A polynomial kernel function changes the structure of a dataset and may cause incorrect clustering, while the Gaussian kernel function is only a radial expansion of the Euclidean measure, which does not change the relative position of the data. Therefore, the Gaussian kernel function is selected, which is used more often. The characteristic space of the Gaussian kernel function has infinite dimensions and the finite sample must be linearly separable in the infinite dimensional space. Equation (8) shows the Gaussian kernel function.

(8) $$k\left(x,y\right)=\exp\left(-\frac{{\left|\left|x-y\right|\right|}^{2}}{2\sigma^{2}}\right),\sigma \in R$$

Therefore, the similarity measure based on the Gaussian kernel is adopted. Then, k(x,x)=1; therefore, Equation (7) can be simplified as follows:

(9) $$d_{H}\left(x,y\right)=\sqrt{2-2k\left(x,y\right)}$$

Currently, kernel techniques are very interesting because there is no need to compute a mapping. If the algorithm can be represented by the inner product between two vectors, then the inner product needs to be replaced with some other suitable space. That is, no matter how the dot product is used, it can be replaced by the kernel function. By using the kernel function, the algorithm can be transformed into a higher-dimensional space instead of mapping the input points to the original space, which is desirable because a high-dimensional feature space can be infinitely dimensional, making it impossible to calculate.

In practical applications, different data have different dimensions. To make data with different units or orders of magnitude comparable, it is usually necessary to make appropriate changes to the data. To ensure the unity of the spatial distance and attribute distance unit of the data, it is necessary to standardize the data in each dimension and then identify the correlation between each data point.

This paper uses the Z-score normalization method, which is applicable to situations when the maximum and minimum values of the attributes are unknown, or the outliers lie beyond the range of values. After normalization, the mean value of each variable is 0 and the standard deviation is 1. The original data are all converted into dimensionless index evaluation values (i.e., the values of each index are at the same quantity level); then, comprehensive evaluation and analysis can be performed.

If $A^{\prime }\;\left(L_{1},{\;B}_{1},S_{1},D_{1},T_{1}\right)$ and $B^{\prime }\;(L_{2},{\;B}_{2},S_{2},D_{2},T_{2})$ represent two standardized data points, including the longitude and latitude data (L and B, respectively), the speed attribute S, the direction attribute D, and the time attribute T (or more attributes), then the distance between the two points is as follows:

(10) $$d_{H}\left(A^{\prime },B^{\prime }\right)=\sqrt{2-2k\left(A^{\prime },B^{\prime }\right)}$$

(11) $$\left|\left|{\;A}^{\prime }-B^{\prime }\right|\right|=\sqrt{{\left(L_{1}-L_{2}\right)}^{2}+{\left(B_{1}-B_{2}\right)}^{2}+{\left(S_{1}-S_{2}\right)}^{2}+{\left(D_{1}-D_{2}\right)}^{2}+{\left(T_{1}-T_{2}\right)}^{2}}$$

Among them, $k\left(A^{\prime },B^{\prime }\right)=\exp\left(-\frac{{\left|\left|A^{\prime }-B^{\prime }\right|\right|}^{2}}{2\sigma^{2}}\right)$, and $\left|\left|{\;A}^{\prime }-B^{\prime }\right|\right|$ represents the Euclidean distance between the spatial location attributes and other nonspatial attributes among the standardized data points.

The clustering process of the AP algorithm is based on the similarity matrix among data. The standard kernel spatial distance is used to replace the Euclidean distance measure of the original algorithm. The corresponding equations for *r* and *a* are changed.

### 3.3. The P of the Adapted Step Length

In the traditional AP algorithm, the preference parameter *P* is set as the mean or median value of the similarity, which can obtain definite clustering but not necessarily the best clustering. According to the principle of the AP algorithm, when the *P* value for each data point is the same, the number of clusters increases as the *P* value increases; therefore, to obtain different clustering numbers, the *P* values of equal distance are obtained within the range $\left[P_{min},P_{max}\right]$ [30] (i.e., a clustering method of adaptive step length and dynamic adjustment of the *P* value). The relevant equations are as follows:

(12) $$P_{min}=\underset{i\neq j}{\min}\;s\left(x_{i},x_{j}\right)$$

(13) $$P_{max}=\underset{i\neq j}{\max}\;s\left(x_{i},x_{j}\right)$$

(14) $$P=\left\{P_{i}|P_{min}+\frac{P_{max}-P_{min}}{M-1}\times \left(i-1\right),i=1,2,\ldots ,M\right\}$$

In Equation (14), *M* represents the input parameter, which means setting up *M* with different *P* values. The analysis of the first equation shows that when i is equal to 1, $P_{i}=P_{min}$; when i is equal to M, $P_{i}=P_{max}$; therefore, the settings of the equations are reasonable. *P* affects the number of clusters, and the number of clusters affects the evaluation index. That is, different *P* values have different evaluation results. Therefore, this paper selects the Davies-Bouldin (DB) index to evaluate the clustering results and then determines the final *P* value according to the evaluation results.

### 3.4. Evaluation Method

Because clustering analysis is an unsupervised algorithm that is unable to determine the best number of categories, many scholars have done research regarding the optimal number of clusters [31,32]. In this paper, the DB index is used to evaluate the effectiveness of the clustering results.

The DB index was proposed by Davies and Bouldin [33]. The main idea is that a reasonable clustering result should be homogeneous and tight within the cluster, and there should be good separation between the clusters. The equation is as follows:

(15) $$DB\left(k\right)=\frac{1}{k}\overset{k}{\underset{i=1}{\sum }}\underset{j=1,\ldots ,k,j\neq i}{\max}\left(\frac{w_{i}+w_{j}}{C_{ij}}\right)$$

where $C_{ij}=\|v_{i}-v_{j}\|$ represents the dispersion degree between the clusters of $C_{i}$ and $C_{j}$; $w_{i}=\frac{1}{\left|C_{i}\right|}\sum_{x\in C_{i}}\|x-v_{i}\|$ represents the average dispersion degree in the cluster i; $v_{i}$ and $v_{j}$ represent the centroids of the clusters $C_{i}$ and $C_{j}$, respectively; $\left|C_{i}\right|$ represents the number of data points in the cluster $C_{i}$; and k represents the total number of clusters. Obviously, when $C_{ij}$ is larger and $w_{i}$ and $w_{j}$ are smaller, the DB value is smaller, and the clustering effect is better. The *k* corresponding to the smallest DB value represents the best clustering.

### 3.5. Pseudocode of the MDST-AP Algorithm

The explanation of pseudocode is as follows: line 1–4 corresponds to the initialization, line 5 corresponds to the normalization, line 7–8 corresponds to the similarity calculation, line 10 corresponds to the responsibility and availability calculation, and line 13 corresponds to the DB index calculation (Algorithm 1).

 **Algorithm 1:**
*MDST-AP* algorithm Input: $X=\left\{x_{1},x_{2},\ldots ,x_{N}\right\}\;:$ Set of objects (dataset of multidimensional attributes) σ: Gaussian kernel parameter (in Equation (8)) *M*
: the number of cluster parameters *P* (in Equation (14)) Output: C =
$\left\{C_{1},C_{2},\ldots ,C_{K}\right\}\;:$ Set of clusters (*X* is divided into *k* clusters)
Let r(i,j)=0, a(i,j)=0    //responsibility and availability λ = 0.5       //the damping coefficient (0<λ<1)*maxits* = 1000    //maximum number of iterations*Convits* = 100     //continuous invariance times of clusters$X^{\prime }$= (*X- X_mean*)/*X_std*       //*X* is normalized to obtain a new dataset $X^{\prime }$ by Z-score //normalization methodFor t = 1 to *M*    //the *M* clustering results are obtained and the best clusters are determined//by using the DB index $S_{N\times N}$ = ComputeSimilarity($X^{\prime \prime }$)   //similarity matrix $S_{N\times N}$ is calculated by Equation (1) and Equation (9)*P* = ComputPreference($S_{N\times N}$, λ)  //preference *P* is optimized by Equation (14)If *maxits* <= 1000 and *Convits* <= 100 Thenr(i,j) and a(i,j) are calculated by Equation (2) and Equation (3)else   //when clustering reaches 1000 iterations, or the cluster center continues 100 //unchanged the final clustering result is reached*clusters* = ComputeCluster(*r* + *a*, $X^{\prime }$)   //the cluster center is determined according to *r* + *a**DB_Index* is calculated by Equation (15)End IfEnd For


### 3.6. Reliability and Complexity Analysis

This paper used the open datasets provided by University of California Irvine (UCI) [34] to analyze the reliability and complexity of MDST-AP algorithm. Table 1 reports some important characteristics of these datasets. To facilitate comparisons of different algorithms, some data are intercepted according to the proportion of each category. Since the class labels were provided for each data, the F-measure method [35] was adopted for clustering evaluation. The larger the F-measure value, the better the clustering effect and the more accurate the algorithm. The comparative experiment of AP algorithm and MDST-AP algorithm was implemented in Python 3.6. The experimental hardware environment includes a 2.8-GHz Intel core i7 CPU, a 500-GB hard disk, and 4.0 GB of memory.

Table 2 presents the average F-measure values and average computational time of the AP and MDST-AP clustering algorithms on the four datasets. In general, the MDST-AP algorithm obtains better clustering results than the AP algorithm in a relatively short time. The average clustering accuracy of the MDST-AP algorithm is 85% and the average computational time is 4.31 seconds. Compared with AP algorithm, the clustering accuracy of the MDST-AP algorithm is improved by 5.5%. The average running time of the MDST-AP algorithm does not exhibit much difference from that of the AP algorithm on a small volume and simple structure dataset (e.g., Iris and Seeds). However, with the increase in dataset volume and the complexity of dataset structure, the operation speed of the MDST-AP algorithm becomes much quicker than that of the AP algorithm. For example, for the Wine quality, white dataset, the average operation time of the MDST-AP algorithm is 3.31 seconds less than that of the AP algorithm.

The complexity of the MDST-AP algorithm is mainly determined by the time required to build the similarity matrix and perform AP clustering. The time complexity of the similarity matrix for all attributes in the kernel space is represented by $O\left(n^{2}\right)$. In addition, the time complexity for running the AP algorithm depends on the number of iterations. Therefore, the time complexity of the whole algorithm is not greater than that of the maximum iteration number for AP clustering, and the minimum time is not less than $O\left(n^{2}\right)$. Generally, the algorithm does not usually achieve the maximum number of iterations unless the algorithm does not converge. Although MDST-AP spends more time than the AP algorithm to calculate the distance matrix, the new similarity measure adopted in the MDST-AP algorithm can better reflect the correlations among the data, which reduces the number of iterations in the operation. Therefore, the entire time required for the algorithm may be reduced. From Table 2, it can be summarized that the MDST-AP algorithm is superior to the AP algorithm in terms of clustering accuracy and speed. 

## 4. Experiments and Analysis

To verify the effect of the algorithm based on the multidimensional attribute data in the kernel space, the MDST-AP algorithm is compared with the original AP algorithm and the results for traffic congestion clustering are analyzed.

### 4.1. Study Area and Data Description

This paper uses 21 days of Global Positioning System (GPS) data for Beijing taxis in November 2012 for the cluster analysis. The total number of GPS records is greater than 218 million. These data are stored in a .txt format at a speed of approximately one file per minute. The main records from the data include taxi ID, recording time, longitude, latitude, vehicle speed, driving direction, and status (0 for empty, 1 for passengers), as indicated in Table 3.

For the research purpose and quality of data, it is necessary to preprocess the data, select the passenger records (i.e., the status is 1) and remove the time errors or record the problematic data to improve the accuracy of the calculation speed and results. By considering the modifiable areal unit problem (MAUP) [36], streets are chosen as the spatial analysis unit; they are selected according to the level and width of the roads. The points of the trajectories are matched over the nearest streets [37], and the coordinate of the point is changed accordingly. After data processing, 5784 streets and approximately 25 million GPS points were collected.

**Figure 2:**
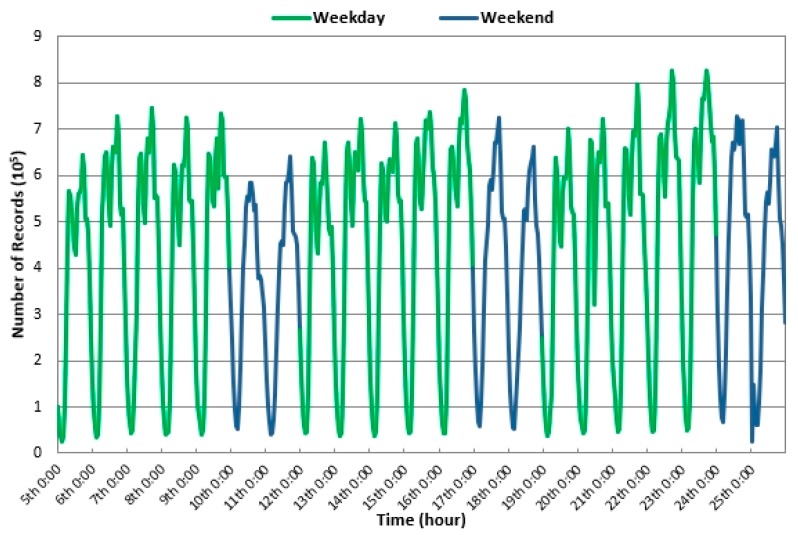
The number of Global Positioning System (GPS) records in three weeks.

The traffic flow in each section of the city varies steadily within a short period of time. The smaller the time section is the more accurate and detailed the description of the change in the dynamic traffic state; however, the frequency of the calculation becomes greater. According to the comprehensive consideration of the amount and accuracy of the data, this paper divides the time period into one-hour intervals [24]. Figure 2 shows the change in taxi point data during three weeks from November 5th to 25th.

The following results can be observed from Figure 2.

1. From Monday to Friday, the tendency in the number of GPS records varies over time during the day but is similar at the same time points on different days. The number of GPS records ranges from 9.14 million to 12.48 million per day on weekdays and the average travel volume is approximately 0.45 million per hour. This result indicates that people’s trip times are essentially the same at a given time on weekdays. There are two distinct peaks on weekdays, namely 8:00 and 17:00.
2. On Saturday and Sunday, the tendency in the number of GPS records varies over time during the day but is similar at the same time points on different days. The number of GPS records ranges from 8.44 million to 10.87 million per day on weekends and the average travel volume is approximately 0.39 million per hour. This result indicates that people’s trip times are essentially the same at a given time on weekends. Overall, the demand for taxis on weekends is lower than that on weekdays. There are also two distinct peaks on weekends, namely 10:00 and 17:00. Regardless of whether it is a weekday or weekend, the minimum number of taxi records in a day occurs from 3:00 to 4:00.

In general, people have different travel habits on weekdays and weekends. On weekends, people travel later and stay out longer than they do on workdays. On weekdays, people have to go to work or school, but on weekends people go out for different types of entertainment. These activities have different effects on urban road traffic.

Based on the analysis of the change trend in taxi trajectories over three weeks, the data are divided into weekdays and weekends. At the same time, based on the morning and evening peak periods, six days of data (three working days on the 7th, 14th, and 22nd, and three rest days on the 11th, 17th, and 25th) with the same daily variation patterns were selected for analysis; the study area was located in the Second Ring Road of Beijing.

### 4.2. Clustering Results and Discussion

According to the analysis of the change trend in taxi trajectory data per hour, this paper selected 8:00 and 17:00 data from three working days and 10:00 and 17:00 data from three resting days for the clustering analysis and then compared and analyzed traffic conditions during the morning and evening peaks on weekdays and weekends. In addition, the similarities and differences in the traffic congestion points during the evening peak are studied. The MDST-AP algorithm was implemented in Python 3.6. The experimental hardware environment includes a 2.8-GHz Intel core i7 CPU, a 500-GB hard disk, and 4.0 GB of memory.

Based on the clustering steps in Section 3.5, this paper used the AP algorithm and the MDST-AP algorithm to perform clustering using the same data and parameters. Generally, only one attribute is considered in the clustering, although to ensure its comparability, the AP algorithm computes the Euclidean distance of multiple attributes (i.e., position, speed, and direction attributes), and then the MDST-AP uses the Gaussian kernel function to simultaneously calculate the similarities among the attributes of position, speed, and direction (using the same time data for comparison, so the time attribute is not considered in the experiment.). The MDST-AP algorithm has different clustering times according to the *M* selection. Based on the optimization formula, the clustering results with the lowest DB index are extracted. The results are presented in Table 4.

Table 4 reports the clustering results of the AP and MDST-AP algorithms in the morning and the evening peak of the three working days. According to Table 4, the clustering results of the AP algorithm have DB index and clustering results that are approximately 10 and 5 times greater than those of the MDST-AP algorithm, respectively, which leads to considerable redundancy. The essence of clustering is to divide the categories with the same conditions using a large amount of data. When it is applied to a road congestion analysis, the number of clusters should be minimized. Large similarities among clusters and large difference between clusters is beneficial to the analysis of specific congestion points. The experimental results reveal no evidence that a greater number of clusters corresponds to a smaller DB index (e.g., the cluster numbers of the MDST-AP algorithm are 18 and 15, and the corresponding DB indexes are 7.84 and 13.23, at 8:00 in Day2 and Day 3, respectively), so the number of clusters and the DB index are not linear. Therefore, when judging the relatively better clustering between the AP algorithm and MDST-AP algorithm, the DB index can be used instead of only the number of clusters. From the comparison experiment, the AP algorithm obtains much worse results than the MDST-AP algorithm. When analyzing a large amount of traffic data, it is not conducive to congestion detection if the number of clusters is too large, and the workload of artificial judgment will increase. In summary, compared to the Gaussian kernel distance of the MDST-AP algorithm, the AP algorithm cannot obtain an ideal clustering result from the Euclidean distance. The MDST-AP algorithm uses the position, speed, and direction attributes at the same time, which greatly improves the availability and reliability of the results. The effectiveness of the MDST-AP algorithm is illustrated by the GPS data clustering experiment.

In the above experiments, to compare the MDST-AP algorithm with the original AP algorithm, the damping coefficient is set at 0.5. However, when the value of λ increases (0.8 and 0.9), the speed of the algorithm that achieves the best clustering increases. The clustering results for the 7th day of data illustrate the convergence speed of the MDST-AP algorithm under different λ values. The main results are reported in Table 5.

From Table 5, it can be observed from the experimental results regarding the minimum DB index under different λ conditions that the DB index of the MDST-AP algorithm reaches a minimum at an *M* value of 3 when λ is 0.5, an M value of 2 when λ is 0.8, and an M value of 1 when λ is 0.9. The results show that the greater the λ value is, the faster the convergence speed of the algorithm; however, a λ value that is too large causes the algorithm to miss the best clustering result. Therefore, the MDST-AP algorithm obtains ideal clustering results quickly when λ is set to 0.8. The results for an M value of 5 under different λ values are the same, which indicates that this M value is not related to the λ value. Therefore, λ is set to 0.8 in this paper to analyze traffic congestion.

**Figure 3:**
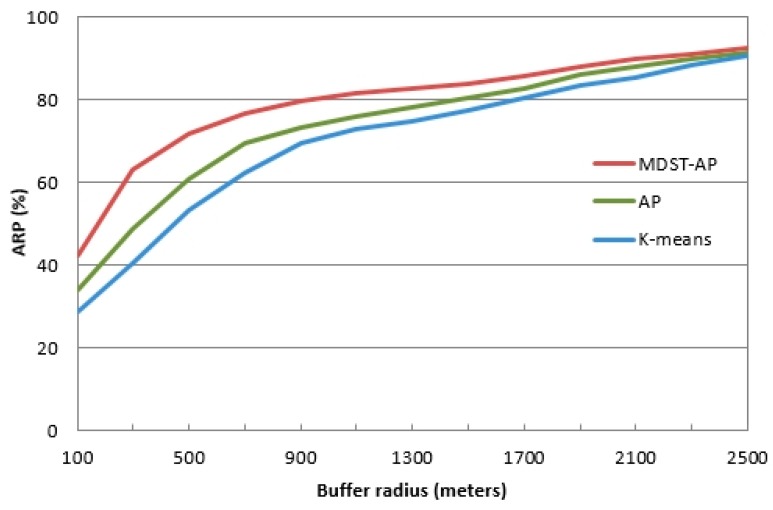
Average ARP of different buffer radii of the k-means, AP, and MDST-AP algorithms.

The data from the 13th day are used to examine the clustering results on a working day to measure the accuracy of the k-means algorithm, the AP algorithm and the MDST-AP algorithm. According to the provisions of the Ministry of Public Security in the “Evaluation Index System of Urban Road Traffic Management”, the average speed of motor vehicles on urban main roads is greater than or equal to 30 kilometers per hour for unimpeded traffic, 20–30 kilometers per hour for mildly congested, 10–20 kilometers per hour for congested traffic, and less than 10 kilometers per hour for very congested traffic. Therefore, based on the speed of each point, the data at 17:00 on the 13th are divided into four clusters to measure the accuracy of the three algorithms. In addition, the clustering results of the evening peak at 17:00 on the three weekdays are also divided into four clusters based on the average speed of each cluster. In this study, the accuracy ratio of the points matched (ARP), which is a commonly used index in the literature [38], was employed to quantify the MDST-AP algorithm accuracy; the ARP is given by:

(16) $$ARP=N_{correct}/N_{original}\times 100\%$$

where $N_{correct}$ represents the number of correctly matched points and $N_{original}$ represents the total number of points in the original GPS data. Within a certain radius, a point must belong to the same cluster of as that of the three workday results, which is the correct clustering point. For example, a very congested point within a 1000-meter buffer zone contains three very congested points, which at least come from the clustering results on different workdays. For the comparability of the algorithms, the conclusions in Table 5 are used. The clustering number of the k-means algorithm is set to 17 and the Euclidean distance of all attributes is calculated as the similarity parameter. The experimental parameters of the AP algorithm and MDST-AP algorithm are specified Table 4. Table 6 presents the clustering accuracy of the k-means algorithm, the AP algorithm and the MDST-AP algorithm within a 1000-meter buffer radius, and a comparison between the data on the 13th day and the weekday clustering results under the same time conditions was performed. Figure 3 shows the average ARP values of the four clusters for different buffer radii.

Table 6 indicates that the average ARP values for the k-means algorithm, AP algorithm, and the MDST-AP algorithm in the 1000-meter buffer zone reach 72.51%, 75.18% and 81.47%, respectively. That is, the ARP of the MDST-AP algorithm is approximately 9% and 6% greater than that of the K-means algorithm and the AP algorithm, accordingly. These results can also be observed in Figure 3. The ARP values of the unimpeded, congested and very congested categories are up to 83% for the K-means algorithm, the AP algorithm, and MDST-AP algorithm; however, the ARP value is very low in the mildly congested category, which is mainly because the number of mildly congested points is very small (only approximately 10% of the total) and the number of points that meet the accuracy requirements is much less. Therefore, the accuracy is relatively low and a mildly congested point can easily become a congested or unimpeded point. On the whole, the accuracy of the MDST-AP algorithm is better than that of the k-means algorithm and the AP algorithm in four congestion categories. In particular, an accuracy of more than 98% is achieved within a one-kilometer radius, which indicates that the MDST-AP algorithm is more accurate in predicting very congested roads. Therefore, the MDST-AP algorithm can be applied to urban road congestion calculations.

Figure 3 shows that the average ARP values of the three algorithms are closer to 90% within a 2000-meter buffer zone. Discrete points are observed in the experimental data, including temporary parking or other special condition points; therefore, the accuracy cannot reach 100%. Generally, the application of the MDST-AP algorithm is relatively more reliable than that of the k-means algorithm and AP algorithm when calculating traffic congestion.

**Figure 4:**
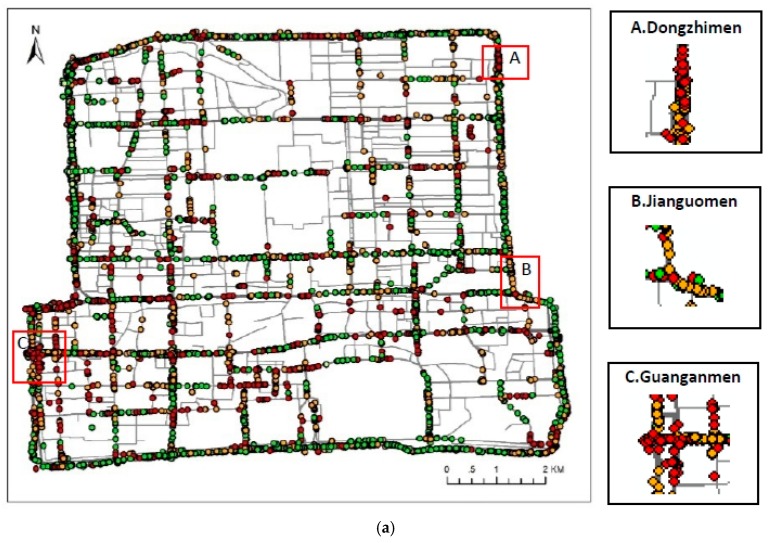

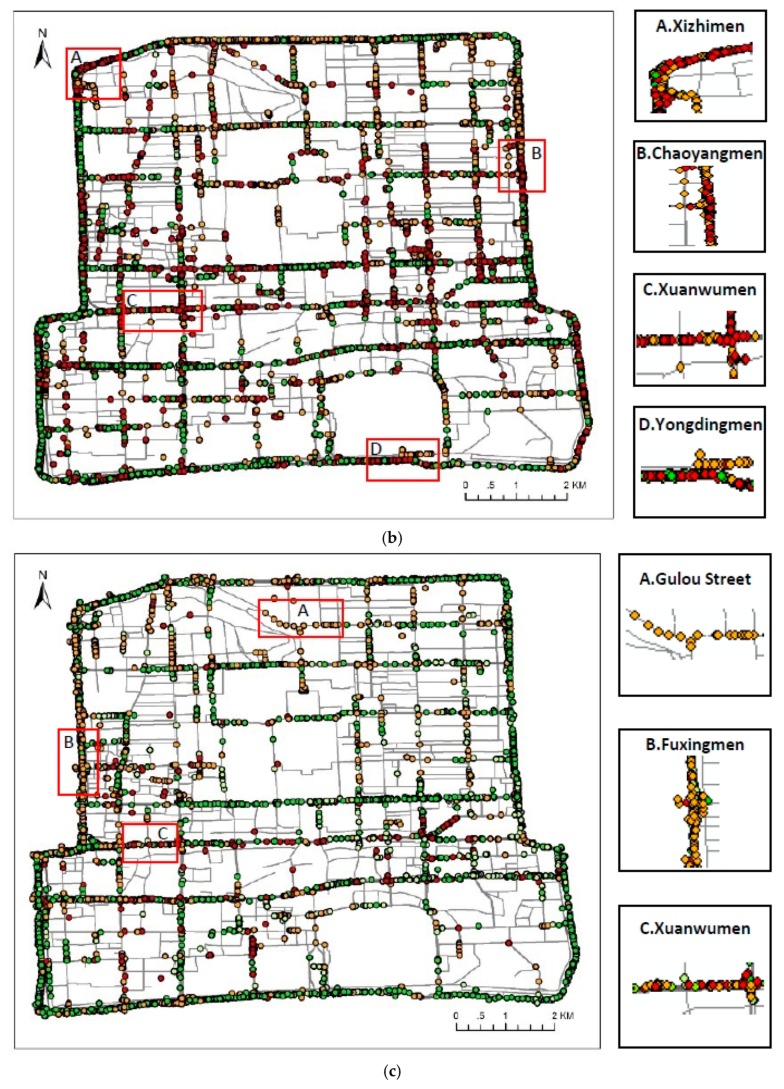

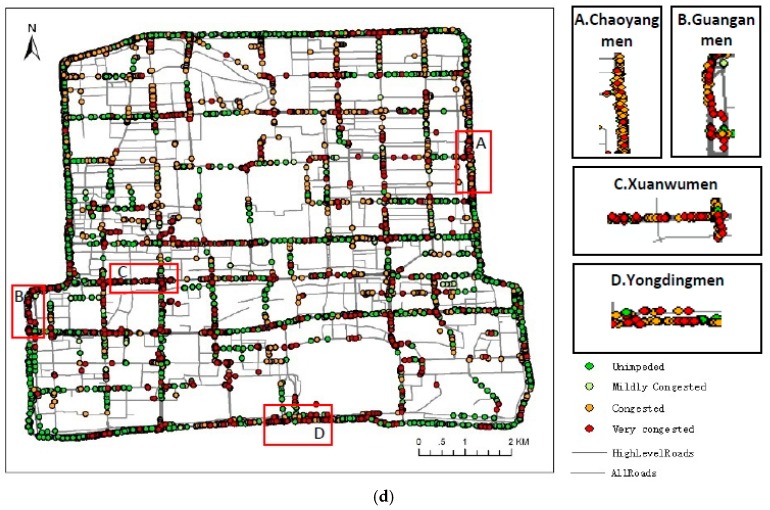
Clustering results of the morning and evening peaks on weekdays and weekends. (**a**) Morning peak on weekdays. (**b**) Evening peak on weekdays. (**c**) Morning peak on weekends. (**d**) Evening peak on weekends.

The MDST-AP algorithm is used to cluster the morning and evening peaks on working and rest days for each of the three analyzed days, and the results were overlapped to compare the traffic conditions between weekdays and weekends during the morning and evening peaks. The size of the trajectory points was adjusted to display the traffic congestion more clearly, which led to the overlap of different points. Therefore, the points seem to be less than they actually are. Based on the average speed of each cluster, the clustering results are divided into four clusters by the Ministry of Public Security to measure the degree of traffic congestion. There are 4958 points in Figure 4a and 6748 points in Figure 4b, which show the clustering results of the morning and evening peaks on weekdays. Likewise, there are 4732 points in Figure 4c and 5682 points in Figure 4d, which show the clustering results of the morning and evening peaks on weekends.

According to the clustering results of the morning peak at 8:00 on weekdays in (a), Guanganmen, Dongzhimen and Jianguomen experience more congestion. According to the clustering results of the evening peak at 17:00 on weekends in (b), Xizhimen, western Xuanwumen, eastern Yongdingmen, and Chaoyangmen (North and South Street) experience more congestion.

According to the clustering results of the morning peak at 10:00 on weekends in (c), Fuxingmen, western Xuanwumen, and Gulou Street experience more congestion. According to the clustering results of the evening peak at 17:00 on weekends in (d), Guanganmen, western Xuanwumen, Yongdingmen, and Chaoyangmen (North and South Street) experience more congestion.

Generally, traffic on weekends near Xizhimen and Dongzhimen is more unimpeded than that on weekdays; Chaoyangmen and Yongdingmen experience congestion during the evening peak on both weekdays and weekends, while Xuanwumen and Guanganmen experience greater congestion every day. These conditions are related to different land use types and individuals’ travel purposes.

According to Table 7, the proportions of traffic congestion on weekdays and weekends are both greater than 70%, indicating that morning and evening peak congestion in the Beijing Second Ring is more severe, and the overall traffic situation on weekends is better than that on weekdays.

## 5. Conclusions

When clustering large spatial data via the AP algorithm, there are some problems, such as considering one attribute only, a large number of clustering results, substantial redundancy, and linear inseparability. Therefore, the MDST-AP algorithm, which is based on multidimensional attributes in a kernel space, is proposed. The Gaussian kernel method is used to solve the problem of the linear inseparability of multidimensional data. The similarity (*P*) value adopted the adaptive step size method, which reduces the number of iterations, improves the convergence speed, and reduces the running time of the algorithm. The experiments of the open datasets demonstrate the efficiency of the MDST-AP algorithm. Through a contrasting experiment between the AP clustering algorithm and MDST-AP algorithm, the AP algorithm has DB index and clustering results that are approximately 10 and 5 times greater than those of the MDST-AP algorithm. The MDST-AP algorithm shows more advantages in big data clustering, which obtained satisfactory results faster by optimizing the value of λ. The results of the accuracy test show that the *ARP* for traffic congestion via the MDST-AP algorithm is more than 81% within the 1000-meter buffer radius, which indicates that the MDST-AP algorithm is more reliable for traffic congestion analysis than the k-means algorithm and AP algorithm. On the whole, the MDST-AP algorithm yields faster and more effective clustering results than the other compared algorithms for multidimensional spatiotemporal datasets.

The MDST-AP algorithm is used to cluster taxi trajectory data in Beijing and calculate the congestion of roads in different directions, which can provide a real-time congestion evaluation and reference for the travel of citizens. Through this case study, the average proportion of traffic congestion is found to be greater than 70% every day, which indicates that the morning and evening peak congestion in the Beijing Second Ring is more serious, and the overall traffic situation on the weekends is better than that on weekdays. Based on the attribute correlations for multiple dimensions, the MDST-AP algorithm can treat large datasets in a shorter time and obtain more ideal results.

In future studies, taxi data will be combined with other spatiotemporal data (such as bus data, mobile signaling data, shared bicycle data, etc.) to improve the accuracy of the congestion calculation of the MDST-AP algorithm. Meanwhile, a parallel computing framework (e.g. Spark) can be applied to enhance the efficiency of the MDST-AP algorithm. In addition, the congestion situation calculated by the MDST-AP algorithm can be displayed on commercial maps (such as Google Map and Baidu Map), which will provide services for travelers and traffic planners.

## Figures and Tables

**Table 1:** University of California Irvine (UCI) datasets information.

Dataset	Iris	Seeds	Wine Quality, Red	Wine Quality, White
Objects	150	150	150	500
Clusters	3	3	6	7
Attributes	4	7	11	11

**Table 2:** Clustering results and computational time for different datasets. AP: affinity propagation.

Algorithms	Measures	Iris	Seeds	Wine Quality, Red	Wine Quality, White
AP	F-measure	0.88	0.81	0.71	0.78
	Time (s)	0.38	0.44	0.54	19.29
MDST-AP	F-measure	0.93	0.89	0.76	0.82
	Time (s)	0.35	0.45	0.47	15.98

**Table 3:** Global Positioning System (GPS) dataset information.

ID	Time	Latitude	Longitude	Speed (km/h)	Direction	Status
174853	20121101001447	116.4548645	39.9519463	51	328	1
453468	20121102155618	116.2787857	39.9250107	25	180	0

**Table 4:** Comparison of algorithms.

		Day 1		Day 2		Day 3	
		Clusters	DB	Clusters	DB	Clusters	DB
8:00	AP	82	116.18	80	129.81	84	128.85
	MDST-AP	18	14.51	18	7.84	15	13.23
17:00	AP	82	129.23	82	124.45	74	190.65
	MDST-AP	18	7.51	14	6.80	10	9.77

**Table 5:** Clustering results under different λ values.

	M	1	2	3	4	5
λ						
**0.5**	Clusters	13	13	**18**	18	998
	DB	11.46	9.57	**7.51**	28.03	29.84
0.8	Clusters	16	**17**	20	24	998
	DB	6.89	**5.47**	13.44	7.23	29.84
0.9	Clusters	**21**	23	24	28	998
	DB	**5.73**	19.62	9.78	6.37	29.84

**Table 6:** Accuracy ratio of the points matched (ARP) comparison of the K-means, AP, and MDST-AP algorithms (%).

Cluster	Unimpeded	Mildly Congested	Congested	Very Congested
K-means	87.54	28.3	83.11	91.07
AP	90.6	31.62	85.3	93.2
MDST-AP	97.12	40.18	90.36	98.2

**Table 7:** Comparison of traffic conditions between weekdays and weekends (%).

Cluster	Unimpeded	Mildly Congested	Congested	Very Congested
Weekdays	25.01	1.47	37.95	35.57
Weekends	29.31	10.89	39.15	20.65
